# Association of sodium-glucose cotransporter 2 inhibitors with post-discharge outcomes in patients with acute heart failure with type 2 diabetes: a cohort study

**DOI:** 10.1186/s12933-023-01896-3

**Published:** 2023-07-28

**Authors:** Sohee Park, Han Eol Jeong, Hyesung Lee, Seng Chan You, Ju-Young Shin

**Affiliations:** 1 School of Pharmacy, Sungkyunkwan University, Suwon, Republic of Korea; 2 Research Department of Practice and Policy, School of Pharmacy, University College London, London, UK; 3 Department of Biohealth Regulatory Science, Sungkyunkwan University, Suwon, Republic of Korea; 4 Department of Biomedical Systems Informatics, Yonsei University College of Medicine, Seoul, Republic of Korea; 5 Department of Clinical Research Design & Evaluation, Samsung Advanced Institute for Health Sciences & Technology, Sungkyunkwan University, Seoul, Republic of Korea

**Keywords:** Acute heart failure, Sodium-glucose cotransporter 2 inhibitors, Postdischarge Outcome, Type 2 diabetes

## Abstract

**Background:**

Given the cumulative evidence on the effectiveness of sodium-glucose cotransporter 2 inhibitors (SGLT2is) on chronic heart failure, demand is emerging for further information on their effects in patients who are hospitalized for acute heart failure. However, there is still limited evidence about the class effect of SGLT2is on acute heart failure. We investigated whether initiating treatment with SGLT2is after an episode of acute heart failure reduces the risks of post-discharge heart failure readmission or cardiovascular mortality among patients with type 2 diabetes.

**Methods:**

A retrospective cohort study was conducted in a cohort of patients with type 2 diabetes who hospitalized for heart failure, using Korean Health Insurance Review & Assessment database (2015–2020). The exposure was defined as initiation of SGLT2is during hospitalization or at discharge. We assessed hazards of post-discharge heart failure readmission and cardiovascular death at 1-year, and 30-, 60-, and 90-day from the date of discharge in the SGLT2is users and non-users. Cox proportional hazards models with propensity score-based inverse probability of treatment weighting were used to estimate hazard ratios and 95% confidence intervals.

**Results:**

Among 56,343 patients with type 2 diabetes hospitalized for heart failure, 29,290 patients were included in the study cohort (mean [SD] age, 74.1 [10.8] years; 56.1% women); 818 patients (2.8%) were prescribed SGLT2is during index hospitalization or at discharge. Patients with a prescription for SGLT2i vs. those without prescription had lower rates of heart failure readmission or cardiovascular death at 1 year (22.4% vs. 25.3%; adjusted hazard ratio, 0.90 [95% confidence interval, 0.87–0.93]), and also at 30 days (7.0% vs. 7.7%%; 0.74 [0.69–0.79]).

**Conclusions:**

Among patients with type 2 diabetes, initiating SGLT2i treatment after an episode of acute heart failure was significantly associated with a reduced combined risk of heart failure readmission and cardiovascular mortality in a nationwide cohort reflecting routine clinical practice.

**Supplementary Information:**

The online version contains supplementary material available at 10.1186/s12933-023-01896-3.

## Background

Acute heart failure (HF) is a common cause of hospitalization, as well as a life-threatening condition associated with high risks of subsequent hospital readmission and mortality [1, 2]. While management of chronic HF has considerably improved over the past decades, there is relatively limited evidence on available treatment options to improve post-discharge clinical outcomes in patients hospitalized for acute HF [3].

Sodium-glucose cotransporter 2 inhibitors (SGLT2is), initially developed as a medication for glucose control in type 2 diabetes (T2D), have recently emerged as a new therapeutic option for HF [1]. Since three pivotal trials initially showed the benefit of the SGLT2i on reducing HF hospitalization in patients with T2D [4–6], their effects on HF have been intensively investigated and there have been substantial changes in the role of SGLT2is in clinical practice and guidelines. Recent meta-analyses of trials further suggest that SGLT2is reduce the risk of hospitalization for HF and cardiovascular death in patients with HF, irrespective of ejection fraction [7, 8]. In light of the cumulative evidence on the effectiveness of SGLT2is on chronic HF [9], demand is elevated for further evidence on their effects in patients who are hospitalized for acute HF.

Although trials with empagliflozin and sotagliflozin have individually shown meaningful clinical benefits in patients hospitalized for acute HF [10, 11], whether these observed benefits can be extended to the class of SGLT2is remains elusive as there is no available data on dapagliflozin. There is also no evidence on relatively long-term follow-up, beyond immediately after discharge from hospital. Furthermore, given the vulnerability of the patients who hospitalized for HF and their life-threatening condition at admission, participants for trials could be different from real-world, due to the ethics and informed consent process [12]. Thus, there is need to generate additional evidence supporting above viewpoints.

Awaiting the results of ongoing trials with dapagliflozin on acute HF, we conducted a retrospective cohort study using real-world data from claims database to assess whether initiating SGLT2is after an episode of acute HF reduce combined risk of post-discharge HF readmission and cardiovascular death among patients with T2D.

## Methods

### Data source

We used the nationwide health insurance claims data of South Korea (2015–2020), derived from Health Insurance Review & Assessment (HIRA) database (Data No. M20210607316). The HIRA database contains deidentified, longitudinal, individual patient-level data on all healthcare use, including but not limited to diagnoses, procedures, and prescription claims from all settings [13]. Diagnoses are coded according to the International Classification of Diseases, 10th Revision (ICD-10), and the overall positive predictive value of diagnostic codes in claims was 82% when comparing any diagnoses to electronic medical records [14]. Drug prescriptions are coded using the domestic coding system that are based on the drug’s active ingredient, dose, route of administration, and dosage form.

### Study population

**Fig. 1:**
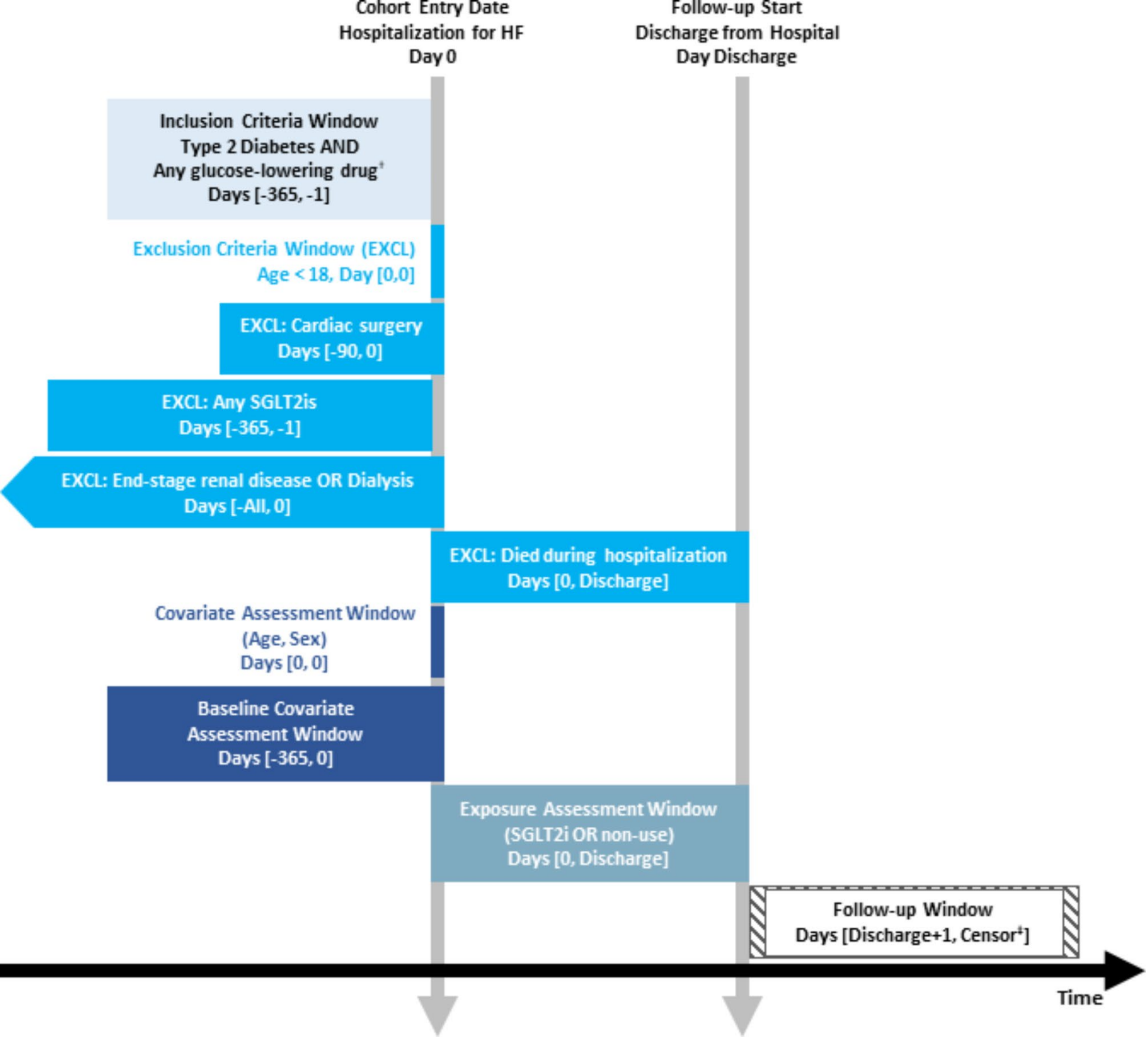
Graphical representation of study design. † Metformin, α-glucosidase inhibitors, GLP-1 receptor agonists, meglitinides, sulfonylureas, dipeptidyl-peptidase 4 inhibitor, or insulin. ‡ Censoring at an earliest of the outcome of interest, death, or 365 days after discharge. Abbreviations: EXCL, exclusion criteria window; HF, heart failure; SGLT2i, sodium-glucose co-transporter 2 inhibitor

We initially identified patients who were hospitalized with HF as a primary or secondary diagnosis between 1 and 2016 and 31 December 2020 among patients with T2D, which were defined as patients who diagnosed with T2D and received pharmacotherapy for T2D within the year before admission. The first date of the hospitalization for HF was defined as a cohort entry. We then excluded patients who (1) were aged < 18 years old at admission, (2) did not receive any antidiabetic treatment within the year before admission, (3) died during the index hospitalization, (4) had a possible post-discharge follow-up period < 1 year to ensure complete 1-year of follow-up to assess outcomes, (5) had a history of cardiac surgery within 90 days before admission, (6) had an end-stage renal disease or dialysis within the year before admission, as a proxy for renal insufficiency (GFR < 45mL/min/1.73m^2^) which is contraindication of SGLT2is, and (7) had a prior prescription for a SGLT2i within the year before admission (see Fig. 1). Detailed diagnostic or procedure codes used to identify the study population are listed in Table S1.

### Exposure

The exposure of interest was the presence of a prescription of a SGLT2i either during the index hospitalization or at discharge; dapagliflozin, empagliflozin, ertugliflozin, and ipragliflozin were included to define exposure. As canagliflozin is not available in South Korea, it was not included the study. The exposure assessment window was defined from the date of hospital admission to the date of discharge from hospital, because the information on exact date of prescription during hospitalization is not available in HIRA database, due to the origin of the database, medical claims bill and the batch billing process for inpatient medical claims. Thus, in this study, exposed group was patients who were prescribe a SGLT2i alone or in combination with other antidiabetic drugs for T2D management during index hospital or at discharge, and comparator group was those not.

### Outcome and follow-up

The primary outcome was defined as composite end points of hospital readmission due to HF or cardiovascular death within a year after discharge. Secondary outcomes were the individual components of the primary outcome. The date of outcome events was defined as the date of hospital admission or in-hospital death with a primary or secondary prespecified diagnoses; diagnostic codes used to define the study outcomes are listed in Table S2. Patients were followed from the date of hospital discharge until the occurrence of a study outcome, in-hospital death, or 365th day of follow-up, whichever occurred first; this follow-up definition was analogous to an intention-to-treat approach. We also performed additional analyses to examine early post-discharge risks: 30-, 60-, and 90-day status of primary and secondary outcomes.

### Propensity score method

To control potential confounding derived from differences between treatment groups, we used the inverse probability treatment weighting (IPTW) approach with asymmetrical trimming [15]. Propensity score, which is the probability of receiving a SGLT2i or not, was estimated using a multivariable logistic regression model that included all potential covariates of age, sex, comorbidities, comedications, healthcare use, level of antidiabetic treatment, and medications at discharge [16]; the full list and descriptions of covariates appear in Table S3. In light of the study population of patients who hospitalized for HF with comorbid T2D, and the distribution of propensity score between exposed and comparator groups (Figure S1), frailty can be a strong confounder in this study. Thus, we applied asymmetric propensity score trimming to exclude patients who were treated most contrary to prediction to reduce bias due to unmeasured confounders in this situation [17]; thresholds were 1st and 99th percentiles of the propensity score distribution in the SGLT2i and non-use groups, respectively. IPTW was then applied to construct a pseudo-population that represent the overall eligible population and estimate the average treatment effect in the entire population (ATE) with balanced baseline characteristics between exposed and comparator groups.

### Statistical analyses

Baseline characteristics were summarized in the unweighted and weighted populations: frequencies and percentages were calculated for categorical variables; mean and standard deviation were calculated for continuous variables. Covariate balance between SGLT2i and non-use groups was assessed using the absolute standardized mean difference (< 0.1 indicates negligible differences) before and after applying IPTW [18].

We reported the crude number of events, incidence rate (IR) per 100 person-years for each outcome by treatment groups. The log-rank test and Cox proportional hazards regression models were used to evaluate the association between SGLT2i versus non-uses and the primary outcome and secondary outcome of cardiovascular death in the crude and weighted populations [19]. For the secondary outcome of HF readmission, we used Gray’s test proportional subdistribution hazards model of Fine and Gray to account for the competing risk of death [20]. The models to estimate adjusted hazard ratios (HRs) were further adjusted for covariates that remained imbalanced between treatment groups in the weighted population (absolute standardized mean difference ≥ 0.1). Proportional hazard assumptions were evaluated using time interaction term before the survival analysis, and there was no violation of this assumption. All analyses were conducted using the SAS Enterprise Guide version 7.1 (SAS Institute Inc., Cary, NC, USA).

### Subgroup analyses

We subclassified the SGLT2i group into dapagliflozin and empagliflozin and compared each SGLT2i molecule with the non-use group to estimate the molecule-specific effect of SGLT2is on acute HF; this analysis was not possible for ertugliflozin and ipragliflozin given the small number of patients prescribed these drugs (ertugliflozin, 4 [0.5%]; ipragliflozin, 1 [0.1%]. We also performed stratified analyses to investigate potential effect modification by HF status (de novo or decompensated), age (< 65 or ≥ 65), sex, history of chronic kidney disease, and medications dispensed at discharge (renin-angiotensin system inhibitor [RASi], β-blockers, mineralocorticoid receptor antagonists). Interactions were tested using the Wald test for heterogeneity. Propensity scores were re-estimated within each strata and the IPTW with asymmetrical trimming was reapplied for each comparison.

### Sensitivity analyses

We performed several sensitivity analyses to assess the robustness of our main findings. First, we applied a stricter definition for HF readmission by using only the primary inpatient diagnosis of HF. Second, non-use group amongst patients with T2D likely represents a broad severity of the respective condition. Thus, to assess confounding by indication, we applied two alternative comparator groups of: (1) any second-line antidiabetic agents prescribed during hospitalization or at discharge, but excluded those prescribed metformin, sulfonylurea, or insulin as monotherapy, and (2) dipeptidyl peptidase-4 inhibitor alone or in combination with other non-use antidiabetics prescribed during hospitalization or at discharge. Third, we used stricter thresholds of 2.5th and 97.5th, 5th and 95th percentiles of the propensity score distribution when applying asymmetrical trimming. Fourth, we used fine stratification weights (ATE) as an alternative weighting method based on propensity score, rather than IPTW with asymmetrical trimming [21]. Fifth, we conducted a post-hoc sensitivity analysis using the E-value, proposed by Ding and VanderWeele, described previously elsewhere, to assess the how strong a potential effect of unmeasured or unaccounted residual confounding would have to be to disregard an observed association [22].

## Results

### Baseline patient characteristics

**Fig. 2:**
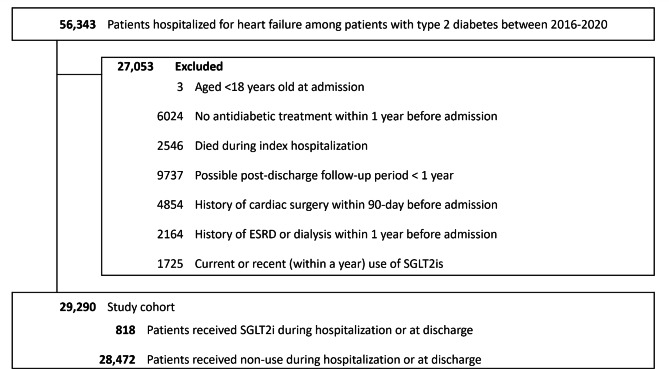
Flow chart describing selection of the study cohort. Abbreviations: ESRD, end-stage renal disease; SGLT2i, sodium-glucose co-transporter 2 inhibitor

There were 56,343 patients with T2D hospitalized for HF in South Korea between 1 and 2016 and 31 December 2020. After applying all exclusion criteria, we identified 29,290 patients for the overall analysis, with a mean age of 74.1 years (standard deviation 10.7) and a higher proportion of females (56.1%). Roughly one third of the study cohort were hospitalized for decompensated HF. Within the study cohort, 818 (2.8%) patients were prescribed a SGLT2i during hospitalization or at discharge (Fig. 2).

The SGLT2i treated patients were younger (mean [SD] age, 69.1 [12.1] years), and more likely to be female (50.4%) and hospitalized for decompensated HF (27.5%) compared with the non-use group. The SGLT2i group generally had a fewer history of comorbidities (e.g., coronary artery disease) and medication use (e.g., calcium channel blockers), despite having a higher proportion of use of HF medications at discharge such as, RASi, mineralocorticoid receptor antagonist, and angiotensin receptor/neprilysin inhibitor. After IPTW with asymmetrical trimming, most covariates were balanced across groups, with absolute standardized mean differences < 0.1; covariates that remained imbalanced were history of chronic kidney disease and use of meglitinides, sulfonylurea, and thiazolidinediones (Table 1; Figure S1, S2).

**Table 1:** Baseline characteristics of patients with type 2 diabetes who hospitalized for acute heart failure

Characteristics^†^	SGLT2i	No SGLT2i	aSD		
	N = 818 (%)	N = 28,472 (%)	Before weighting	After weighting^‡^	
**Age, years**					
Mean (std)	69.1 (12.2)	74.2 (10.7)	0.449	0.027	
**Sex**					
Male	406 (49.6)	12,445 (43.7)	0.119	0.035	
Female	412 (50.4)	16,027 (56.3)			
**Heart failure status**					
Acute de novo	593 (72.5)	19,035 (66.9)	0.123	0.040	
Decompensated	225 (27.5)	9,437 (33.1)			
**Comorbidity**					
Atrial fibrillation	109 (13.3)	4,334 (15.2)	0.054	0.006	
Cancer	65 (7.9)	2,433 (8.5)	0.022	0.070	
Cardiac surgery	23 (2.8)	883 (3.1)	0.017	0.036	
Cerebrovascular disease	58 (7.1)	3,449 (12.1)	0.171	0.060	
Chronic kidney disease	28 (3.4)	3,095 (10.9)	0.292	0.214	
Chronic respiratory disease	198 (24.2)	7,910 (27.8)	0.082	0.037	
Coronary artery disease	157 (19.2)	7,250 (25.5)	0.151	0.025	
Dyslipidemia	289 (35.3)	10,012 (35.2)	0.003	0.053	
Hypertension	493 (60.3)	18,538 (65.1)	0.100	0.048	
Peripheral artery disease	82 (10.0)	3,316 (11.6)	0.052	0.008	
**Comedications**					
Antiplatelets/anticoagulants	543 (66.4)	21,197 (74.4)	0.177	0.034	
Beta-blockers	379 (46.3)	14,700 (51.6)	0.106	0.018	
Calcium channel blockers	401 (49.0)	16,578 (58.2)	0.185	0.006	
Digoxin	109 (13.3)	3,798 (13.3)	0.000	0.026	
Diuretics	464 (56.7)	18,762 (65.9)	0.189	0.019	
Lipid lowering drugs	558 (68.2)	20,804 (73.1)	0.107	0.017	
Nitrates	150 (18.3)	6,745 (23.7)	0.132	0.051	
RASi	556 (68.0)	20,573 (72.3)	0.094	0.035	
ARNi	8 (1.0)	108 (0.4)	0.073	0.013	
**Antidiabetic medications**					
Insulin	222 (27.1)	10,232 (35.9)	0.190	0.015	
Alpha-glucosidase inhibitors	40 (4.9)	1,168 (4.1)	0.038	0.026	
GLP-1 receptor agonists	10 (1.2)	157 (0.6)	0.072	0.044	
Meglitinides	5 (0.6)	407 (1.4)	0.081	0.113	
Metformin	724 (88.5)	22,115 (77.7)	0.292	0.053	
Sulfonylureas	510 (62.3)	15,711 (55.2)	0.146	0.114	
Thiazolidinediones	148 (18.1)	3,675 (12.9)	0.144	0.101	
DPP4i	575 (70.3)	22,112 (77.7)	0.169	0.058	
**Level of antidiabetic treatment** ^**§**^					
1	77 (9.4)	3,382 (11.9)	0.080	0.091	
2	519 (63.4)	14,858 (52.2)	0.230	0.070	
3	222 (27.1)	10,232 (35.9)	0.190	0.015	
**Healthcare use**					
**No. of hospitalizations**					
0	473 (57.8)	13,050 (45.8)	0.242	0.088	
1–2	279 (34.1)	11,473 (40.3)	0.128	0.047	
≥3	66 (8.1)	3,949 (13.9)	0.186	0.063	
**No. of outpatient visits**,					
0–2	6 (0.7)	116 (0.4)	0.043	0.071	
3–5	18 (2.2)	306 (1.1)	0.089	0.014	
≥6	794 (97.1)	28,050 (98.5)	0.099	0.055	
**Medications at discharge**					
RASi	719 (87.9)	23,122 (81.2)	0.186	0.036	
Beta-blockers	163 (19.9)	4,786 (16.8)	0.081	0.035	
MRA	534 (65.3)	12,826 (45.0)	0.416	0.027	
ARNi	39 (4.8)	274 (1.0)	0.230	0.045	

† All covariates, except for medications at discharge, were assessed within a year before cohort entry date, including the cohort entry date

‡ Based on the propensity score, inverse probability of treatment weighting with asymmetrical trimming was applied to balance exposed and comparator groups conditional on measured covariates. This method generated a pseudo population of weighted patients with similar distribution of measured covariates in the exposed and comparator groups

§ Use of antidiabetic drugs past 365 days before the cohort entry date: level 1, only one class of non-insulin antidiabetic medications; level 2, at least two classes of non-insulin antidiabetic medications; or level 3 at least one insulin treatment as alone or in combination with other antidiabetic medications

Abbreviations: ARNi, angiotensin receptor/neprilysin inhibitor; DPP4i, dipeptidyl peptidase-4 inhibitor; HF, heart failure; MRA, mineralocorticoid receptor antagonist; RASi, renin-angiotensin system inhibitor; SGLT2i, sodium-glucose co-transporter 2 inhibitor; std, standard deviation; aSD, absolute standardized difference

### Incidences and hazards of study outcomes

**Fig. 3:**
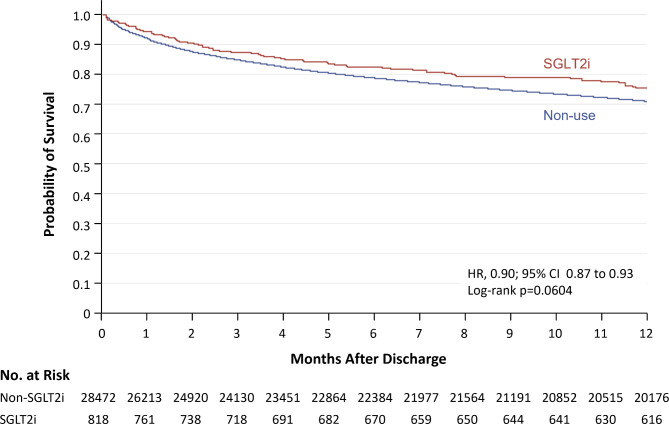
Weighted Kaplan-Meier plot for heart failure readmission or cardiovascular death. Note: Inverse probability of treatment weighting on the propensity score with asymmetrical trimming was used to balance comparison groups on indicators of baseline characteristics. This method produced a weighted pseudo sample of patients in the exposed and reference group with the same distribution of measured covariates. Abbreviations: HR, hazard ratio; SGLT2i, sodium-glucose cotransporter 2 inhibitor

Figure 3 illustrates weighted Kaplan-Meier plot for the primary outcome. The absolute IR in the SGLT2i group was lower than the non-use group at 365 days after discharge: 26.9 versus 31.7 cases per 100 person-years. The risk of HF readmission or cardiovascular death was lower with SGLT2is than non-use (crude HR 0.87, 95% CI 0.75-1.00; weighted HR 0.90, 95% CI 0.87–0.93) although the crude analysis did not show the statistical significance. Effect estimates for the secondary outcomes were similar to that of the primary outcome: crude and adjusted HRs were 0.86 (95% CI 0.74-1.00) and 0.89 (0.86–0.92) for HF readmission and 0.70 (0.42–1.16) and 0.64 (0.57–0.72) for cardiovascular death (Table 2).

**Table 2:** Crude event rates and estimated hazard ratios during the 1-year post-discharge period in patients with type 2 diabetes who hospitalized for acute heart failure

	SGLT2i (N = 818)			Non-use (N = 28,472)			Crude HR (95% CI)	Weighted HR^‡^ (95% CI)
	**Events, N**	**IR** ^†^		**Events, N**	**IR** ^†^			
Composite outcome	183	26.9		7,216	31.7		0.87 (0.75-1.00)	0.90 (0.87–0.93)
HF readmission	176	25.9		6,956	30.5		0.86 (0.74-1.00)	0.89 (0.86–0.92)
Cardiovascular death	15	1.9		746	2.8		0.70 (0.42–1.16)	0.64 (0.57–0.72)

† The number of events per 100 person-years

‡ Inverse probability of treatment weighting on the propensity score with asymmetrical trimming was used to balance comparison groups on indicators of baseline characteristics. This method produced a weighted pseudo sample of patients in the exposed and reference group with the same distribution of measured covariates

Abbreviations: CI, confidence interval; HF, heart failure; HR, hazard ratio; IR, incidence rate; SGLT2i, sodium-glucose co-transporter 2 inhibitor

At 30-, 60-, and 90-days post-discharge, the absolute IRs were approximately 2 to 3-fold higher than those of at 365 days. Before adjusting the baseline covariates, the HRs were 0.90 (95% CI 0.69–1.17) at 30-day, 0.81 (0.64–1.01) at 60-day, and 0.82 (0.67–1.01) at 90-day for the primary outcome. After PS adjustment, HRs were 0.74 (0.69–0.79) at 30-day, 0.82 (0.78–0.86) at 60-day, and 0.84 (0.80–0.88) at 90-day for the primary outcome (Table 3).

**Table 3:** Results of early post-discharge phase analysis

	SGLT2i (N = 818)			Non-use (N = 28,472)			Crude HR (95% CI)	Weighted HR^‡^ (95% CI)
	**Events**	**IR** ^†^		**Events**	**IR** ^†^			
**At 30 day**								
Composite outcome	57	88.5		2,199	98.6		0.90 (0.69–1.17)	0.74 (0.69–0.79)
HF readmission	56	87.0		2,155	96.7		0.90 (0.69–1.18)	0.74 (0.69–0.79)
Cardiovascular death	2	3.0		98	4.2		0.71 (0.18–2.87)	0.58 (0.42–0.81)
**At 60 day**								
Composite outcome	77	61.2		3308	76.6		0.81 (0.64–1.01)	0.82 (0.78–0.86)
HF readmission	74	58.8		3228	74.7		0.79 (0.63-1.00)	0.81 (0.77–0.85)
Cardiovascular death	4	3.0		194	4.2		0.72 (0.27–1.93)	0.56 (0.44–0.72)
**At 90 day**								
Composite outcome	95	51.3		3,980	62.9		0.82 (0.67–1.01)	0.84 (0.80–0.88)
HF readmission	91	49.1		3,877	61.2		0.81 (0.66-1.00)	0.81 (0.77–0.85)
Cardiovascular death	7	3.5		263	3.8		0.93 (0.44–1.96)	0.96 (0.80–1.16)

† The number of events per 100 person-years

‡ Inverse probability of treatment weighting on the propensity score with asymmetrical trimming was used to balance comparison groups on indicators of baseline characteristics. This method produced a weighted pseudo sample of patients in the exposed and reference group with the same distribution of measured covariates

Abbreviations: CI, confidence interval; HF, heart failure; HR, hazard ratio; IR, incidence rate; SGLT2i, sodium-glucose co-transporter 2 inhibitor

### Subgroup and sensitivity analyses

Figure S3-S6 present findings from subgroup analysis. Results for individual SGLT2is showed similar trends of reduced risks for both dapagliflozin (adjusted HR, 0.88; 95% CI, 0.85–0.92) and in empagliflozin (adjusted HR 0.76, 95% CI 0.73–0.79) when compared with the non-use group. Significant effect modifications by following factors were observed: HF status, aged ≥ 65, history of chronic kidney disease, and a prescription of RAS inhibitor, and beta-blocker at discharge with p_interaction_ < 0.001. Results of sensitivity analyses were compatible with the main analysis (Table S5). The E-value was 1.36 for the point estimate and 1.28 for the upper bound of the CI.

## Discussion

This nationwide retrospective cohort study of patients with T2D hospitalized for acute HF suggests that SGLT2i treatment after an episode of acute HF may have post-discharge clinical benefit beyond glucose control. When comparing SGLT2is with non-use, the use of SGLT2is was associated with a lower rate for HF readmission and cardiovascular death both at 1-year and vulnerable phase. Although possible effect modifications were observed, exploratory nature of subgroup analysis requires careful interpretation of the results.

Although statistical significance was not achieved in crude analysis for most outcomes, the HF readmission and cardiovascular mortality rates were consistently lower in the group of patients treated with SGLT2is. Given the recently suggested biological link of SGLT2is with cardioprotective effects [9, 23, 24] and established favorable effect of SGLT2is on chronic HF [25], findings from this study provide a valuable information and indicate a signal of possible beneficial effect of SGLT2i in patients with acute HF. Future studies with larger sample size and a broader population should examine the effect of SGLT2i on post-discharge outcome of acute HF addressing the additional HF-related confounding.

In early post-discharge vulnerable phase analyses, a greater magnitude of effect estimates was observed than main analysis assessing 1-year post-discharge outcome. These findings may be explained by distinctly different mechanisms of action of SGLT2is than other conventional treatments for HF. Effects of SGLT2is are known as less likely to activate neurohumoral pathways; their cardioprotective effects may be multifactorial but mainly by blocking the resorption of filtered glucose in proximal tubules [8, 24]. Osmotic diuresis, which is also differ from loop diuretics, induce electrolyte-free water clearance and greater fluid clearance from the interstitial fluid than blood, and they further contribute to less deleterious effects from excessive blood depletion in decongestion [23]. Their effects on congestion and background cardiovascular benefits indicate potential role of SGLT2is both as a symptomatic and disease modifying therapy to improve clinical outcomes following an episode of acute HF.

After the introduction of SGLT2is as a potential therapeutic option for chronic HF, several observational studies have assessed the effectiveness of SGLT2is on acute HF and findings consistently suggested clinical benefit of SGLT2is [26–29]. A post-hoc analysis of the EMPA-REG OUTCOME trial and an observational study using single-center electronic medical record showed a lower risk of post-discharge HF readmission or cardiovascular death in empagliflozin- and canagliflozin-treated patients, respectively [27, 28]. Two observational cohort studies also showed greater diuretic response in SGLT2i-treated patients via hospitalization [26, 29]. However, these studies were relatively small, and mostly targeted single agent of SGLT2is, making it difficult to draw conclusions for clinical practice.

Focusing on SGLT2is’ potential role to improve post-discharge outcomes in the care of patients hospitalized for acute HF, there have been two completed trials and our findings were in line with these trials. Results from trials have previously demonstrated that initiating SGLT2is after an episode of acute HF led to significant clinical benefits [10, 11]. Empagliflozin, as compared with placebo, showed significant clinical benefits at 90 days after admission (win ratio 1.36; 95% CI, 1.09–1.68) [11]. Although the evidence for dapagliflozin, the most commonly used SGLT2i, is yet to be revealed, the results of DAPA ACT HF trial (NCT04363697) are expected soon to provide significant evidence on clinical practice. Our findings, assessing the effectiveness of all reimbursed SGLT2is in South Korea (dapagliflozin, empagliflozin, ertugliflozin, and ipragliflozin) in real-world, add to existing data by suggesting a potential clinical benefit with this class of agents on acute HF.

There are several challenges in evaluating the effectiveness of a medication using real-world data. First, given the nature of claims data, there might be a significant limitation for investigating questions regarding acute HF; we were unable to evaluate clinical data which are potentially reflective of SGLT2i treatment or of the severity and progression during hospitalization including but not limited to left ventricular ejection fraction, frailty, economic background, New York Heart Association (NYHA) Functional Classification, NT-proBNP, estimated glomerular filtration rate, blood pressure, results of echocardiogram and blood test at admission, and others. The previous meta-analysis demonstrated the benefit of SGLT2is for HF is consistent across the ranges of ejection fraction or NT-proBNP in trial settings [7], but we still cannot rule out the possibility of residual and unmeasured confounding from other factors. Therefore, we attempted to estimate less biased effect estimates by unmeasured confounding by assessing 39 covariates to generate propensity scores and propensity score range restrictions [30]. In light of the lack of clinically important information in this study, our findings should be interpreted carefully with the E-value which indicate the potential impact of unmeasured confounders on the observed association.

Second, there is potential selection bias related to the impact of the progression of kidney disease during admission on being prescribed SGLT2is. We cannot rule out the possibility of a group progressing kidney disease, which may have a poorer condition, tended to be not prescribed SGLT2is and thus, allocated to non-use group. Further studies using clinical and laboratory data are warranted to corroborate our findings. Third, information on exactly when patients initiated SGLT2is during hospitalization was unavailable in the HIRA database. In light of the potential role of SGLT2is as diuretic for decongestion and guideline-directed medical therapy, it is important to optimize the initiation of the SGLT2is in an episode of acute HF, whether early initiation immediately after hospital admission or delayed initiation during hospitalization. Further studies using registries or electronic medical records could possibly answer the question of when it would be optimal to initiate SGLT2is. Fourth, there may have been potential exposure and outcome misclassification due to the nature of retrospective study. However, exposure misclassification, from non-use to SGLT2i group, is likely to the estimates toward the null, and we attempted to minimize outcome misclassification by using a stricter definition with a higher specificity. Fifth, despite the study population with comorbid T2D, we were unavailable to evaluate duration of T2D because it is usually lifelong condition and we can acquire only limited period of data. However, we have addressed the impact of diabetes severity using level of antidiabetic treatment as a for proxy for diabetes severity, which is commonly used in observational studies in T2D [31–34].

Last, due to the nature of the retrospective database study, our study cohort was patients hospitalized for HF among T2D. As SGLT2is were exclusively prescribed for the treatment of T2D during our study period given the local reimbursement criteria in South Korea, we couldn’t define a study population without restriction to diabetic patients for comparability, even though current need for evidence of the effectiveness of SGLT2is is at the patients hospitalized for HF regardless of T2D. This led to relatively small sample size in this study and findings from this study are not likely to be generalizable to patients with HF without T2D. Future studies with larger sample size and a broader population should examine the effect of SGLT2i on post-discharge outcome of acute HF addressing the additional HF-related confounding.

## Conclusions

In conclusion, initiating SGLT2i treatment after an episode of acute HF was significantly associated with a reduced risk of composite outcome of HF readmission and cardiovascular mortality when compared with non-use among patients with T2D. These findings suggest that potential clinical benefit of SGLT2is in the care of patients hospitalized for acute HF. While results from ongoing trials addressing this clinical question are expected to be shared soon, our findings based on real-world data provide valuable evidence for the current context of SGLT2is.

## Electronic supplementary material

Below is the link to the electronic supplementary material.

**Supplements**: Table S1. International Classification of Disease 10th revisions (ICD-10) or procedure codes to identify study population. Table S2. International Classification of Disease 10th revisions (ICD-10) codes to define each outcome. Table S3. List of covariates for generating propensity score. Table S4. SGLT2i treatment before and after weighting. Table S5. Results of sensitivity analyses. Figure S1. Propensity score distributions before and after applying inverse probability treatment weighting with asymmetrical trimming. Figure S2. Plot of standardized mean differences before and after weighting. Figure S3. Results of subgroup and stratified analyses on 1-year post-discharge outcome. Figure S4. Results of subgroup and stratified analyses on 30-day post-discharge outcome. Figure S5. Results of subgroup and stratified analyses on 60-day post-discharge outcome. Figure S6. Results of subgroup and stratified analyses on 90-day post-discharge outcome

## Data Availability

The data that support the findings of this study are available from the Health Insurance Review and Assessment Service of South Korea but restrictions apply to the availability of these data due to domestic laws and regulations that prohibit the distribution or release of individual’s data to the public, and so are not publicly available. Data are however available from the authors upon reasonable request and with permission of the Health Insurance Review and Assessment Service of South Korea.

## List of abbreviations

ATE: Average treatment effect in the entire population
HF: Heart failure
HR: Hazard ratio
IPTW: Inverse probability treatment weighting
IR: Incidence rate
RASi: Renin-angiotensin system inhibitor
SGLT2i: Sodium-glucose cotransporter 2 inhibitors
T2D: Type 2 diabetes

## References

[1] McDonagh TA, Metra M, Adamo M, Gardner RS, Baumbach A, Böhm M (2022). 2021 ESC Guidelines for the diagnosis and treatment of acute and chronic heart failure: developed by the Task Force for the diagnosis and treatment of acute and chronic heart failure of the European Society of Cardiology (ESC). With the special contribution of the heart failure Association (HFA) of the ESC. Eur J Heart Fail.

[2] Arrigo M, Jessup M, Mullens W, Reza N, Shah AM, Sliwa K (2020). Acute heart failure. Nat Rev Dis Primers.

[3] Greene SJ, Fonarow GC, Vaduganathan M, Khan SS, Butler J, Gheorghiade M (2015). The vulnerable phase after hospitalization for heart failure. Nat Rev Cardiol.

[4] Zinman B, Wanner C, Lachin JM, Fitchett D, Bluhmki E, Hantel S (2015). Empagliflozin, Cardiovascular Outcomes, and mortality in type 2 diabetes. N Engl J Med.

[5] Neal B, Perkovic V, Mahaffey KW, de Zeeuw D, Fulcher G, Erondu N (2017). Canagliflozin and Cardiovascular and renal events in type 2 diabetes. N Engl J Med.

[6] Wiviott SD, Raz I, Bonaca MP, Mosenzon O, Kato ET, Cahn A (2019). Dapagliflozin and Cardiovascular Outcomes in type 2 diabetes. N Engl J Med.

[7] Vaduganathan M, Docherty KF, Claggett BL, Jhund PS, de Boer RA, Hernandez AF (2022). SGLT-2 inhibitors in patients with heart failure: a comprehensive meta-analysis of five randomised controlled trials. Lancet.

[8] Jhund PS, Kondo T, Butt JH, Docherty KF, Claggett BL, Desai AS (2022). Dapagliflozin across the range of ejection fraction in patients with heart failure: a patient-level, pooled meta-analysis of DAPA-HF and DELIVER. Nat Med.

[9] Packer M. SGLT2 inhibitors: role in protective reprogramming of cardiac nutrient transport and metabolism. Nat Rev Cardiol. 2023. 10.1038/s41569-022-00824-4 36609604

[10] Bhatt DL, Szarek M, Steg PG, Cannon CP, Leiter LA, McGuire DK (2021). Sotagliflozin in patients with diabetes and recent worsening heart failure. N Engl J Med.

[11] Voors AA, Angermann CE, Teerlink JR, Collins SP, Kosiborod M, Biegus J (2022). The SGLT2 inhibitor empagliflozin in patients hospitalized for acute heart failure: a multinational randomized trial. Nat Med.

[12] Lawrence DS, Ssali A, Moshashane N, Nabaggala G, Maphane L, Harrison TS (2022). Decision making in a clinical trial for a life-threatening illness: therapeutic expectation, not misconception. Soc Sci Med.

[13] Kim JA, Yoon S, Kim LY, Kim DS (2017). Towards actualizing the value potential of Korea Health Insurance Review and Assessment (HIRA) data as a resource for Health Research: strengths, Limitations, applications, and strategies for optimal use of HIRA data. J Korean Med Sci.

[14] Review HI, Service A. Evaluation and consideration methods of consistency between health insurance claims diagnostic codes and medical records. Health Insurance Review and Assessment Service South Korea; 2017.

[15] Austin PC, Stuart EA (2015). Moving towards best practice when using inverse probability of treatment weighting (IPTW) using the propensity score to estimate causal treatment effects in observational studies. Stat Med.

[16] Rosenbaum PR, Rubin DB (1983). The central role of the propensity score in observational studies for causal effects. Biometrika.

[17] Stürmer T, Rothman KJ, Avorn J, Glynn RJ (2010). Treatment effects in the presence of unmeasured confounding: dealing with observations in the tails of the propensity score distribution—a simulation study. Am J Epidemiol.

[18] Austin PC (2009). Balance diagnostics for comparing the distribution of baseline covariates between treatment groups in propensity-score matched samples. Stat Med.

[19] Cox DR (1972). Regression models and life-tables. J R Stat Soc Series B Stat Methodol.

[20] Fine JP, Gray, RJJJotAsa (1999). A proportional hazards model for the subdistribution of a competing risk. J Am Stat Assoc.

[21] Desai RJ, Rothman KJ, Bateman BT, Hernandez-Diaz S, Huybrechts KF (2017). A propensity-score-based Fine Stratification Approach for Confounding Adjustment when exposure is infrequent. Epidemiology.

[22] Ding P, VanderWeele TJ (2016). Sensit Anal Without Assumptions Epidemiol.

[23] Hallow KM, Helmlinger G, Greasley PJ, McMurray JJV, Boulton DW (2018). Why do SGLT2 inhibitors reduce heart failure hospitalization? A differential volume regulation hypothesis. Diabetes Obes Metab.

[24] Joshi SS, Singh T, Newby DE, Singh J (2021). Sodium-glucose co-transporter 2 inhibitor therapy: mechanisms of action in heart failure. Heart.

[25] Zou X, Shi Q, Vandvik PO, Guyatt G, Lang CC, Parpia S (2022). Sodium-glucose Cotransporter-2 inhibitors in patients with heart failure: a systematic review and Meta-analysis. Ann Intern Med.

[26] Griffin M, Riello R, Rao VS, Ivey-Miranda J, Fleming J, Maulion C (2020). Sodium glucose cotransporter 2 inhibitors as diuretic adjuvants in acute decompensated heart failure: a case series. ESC Heart Fail.

[27] Savarese G, Sattar N, Januzzi J, Verma S, Lund LH, Fitchett D (2019). Empagliflozin is Associated with a lower risk of Post-Acute Heart failure rehospitalization and mortality. Circulation.

[28] Martín E, López-Aguilera J, González-Manzanares R, Anguita M, Gutiérrez G, Luque A et al. Impact of Canagliflozin in patients with type 2 diabetes after hospitalization for Acute Heart failure: a Cohort Study. J Clin Med. 2021;10(3). 10.3390/jcm10030505 PMC7867051 33535424

[29] Pérez-Belmonte LM, Sanz-Cánovas J, Millán-Gómez M, Osuna-Sánchez J, López-Sampalo A, Ricci M (2022). Clinical benefits of empagliflozin in very old patients with type 2 diabetes hospitalized for acute heart failure. J Am Geriatr Soc.

[30] Sturmer T, Rothman KJ, Avorn J, Glynn RJ (2010). Treatment Effects in the Presence of Unmeasured Confounding: dealing with observations in the tails of the propensity score Distribution–A Simulation Study. Am J Epidemiol.

[31] Douros A, Rouette J, Yin H, Yu OHY, Filion KB, Azoulay L (2019). Dipeptidyl Peptidase 4 inhibitors and the risk of Bullous Pemphigoid among patients with type 2 diabetes. Diabetes Care.

[32] Yu OHY, Dell’Aniello S, Shah BR, Brunetti VC, Daigle JM, Fralick M (2020). Sodium-glucose cotransporter 2 inhibitors and the risk of below-knee amputation: a Multicenter Observational Study. Diabetes Care.

[33] Faillie JL, Yu OH, Yin H, Hillaire-Buys D, Barkun A, Azoulay L (2016). Association of bile Duct and Gallbladder Diseases with the Use of Incretin-Based drugs in patients with type 2 diabetes Mellitus. JAMA Int Med.

[34] Filion KB, Lix LM, Yu OH, Dell’Aniello S, Douros A, Shah BR (2020). Sodium glucose cotransporter 2 inhibitors and risk of major adverse cardiovascular events: multi-database retrospective cohort study. BMJ.

