# Supplementary material for: Association of sodium-glucose cotransporter 2 inhibitors with post-discharge outcomes in patients with acute heart failure with type 2 diabetes: a cohort study
Source: Cardiovasc Diabetol. 2023 Jul 28;22:191. doi: 10.1186/s12933-023-01896-3 (PMC10386764; doi:10.1186/s12933-023-01896-3)
Supplement: Supplementary file 1 — Supplements: Table S1. International Classification of Disease 10th revisions (ICD-10) or procedure codes to identify study population. Table S2. International Classification of Disease 10th revisions (ICD-10) codes to define each outcome. Table S3. List of covariates for generating propensity score. Table S4. SGLT2i treatment before and after weighting. Table S5. Results of sensitivity analyses. Figure S1. Propensity score distributions before and after applying inverse probability treatment weighting with asymmetrical trimming. Figure S2. Plot of standardized mean differences before and after weighting. Figure S3. Results of subgroup and stratified analyses on 1-year post-discharge outcome. Figure S4. Results of subgroup and stratified analyses on 30-day post-discharge outcome. Figure S5. Results of subgroup and stratified analyses on 60-day post-discharge outcome. Figure S6. Results of subgroup and stratified analyses on 90-day post-discharge outcome [file 12933_2023_1896_MOESM1_ESM.docx]

**Supplements**

Table S1. International Classification of Disease 10th revisions (ICD-10) or procedure codes to identify study population

Table S2. List of International Classification of Disease 10th revisions (ICD-10) codes to define each outcome

Table S3. List of covariates for generating propensity score

Table S4. SGLT2i treatment before and after weighting

TableS5. Results of sensitivity analyses

Figure S1. Propensity score distributions before and after applying inverse probability treatment weighting with asymmetrical trimming

Figure S2. Plot of standardized mean differences before and after weighting

Figure S3. Results of subgroup and stratified analyses on 1-year post-discharge outcome

Figure S4. Results of subgroup and stratified analyses on 30-day post-discharge outcome

Figure S5. Results of subgroup and stratified analyses on 60-day post-discharge outcome

Figure S6. Results of subgroup and stratified analyses on 90-day post-discharge outcome

**Table S1. International Classification of Disease 10^th^ revisions (ICD-10) or procedure codes to identify study population**

|  | **ICD-10 or procedure code** | **Type of diagnosis (primary, secondary)/ type of hospital contact** |
| --- | --- | --- |
| Heart failure admission | I50x | Any / admission |
| Type 2 diabetes | E11x, E12x, E13x, E14x | Any / inpatient or outpatient |
| ESRD or dialysis | N185, Z49x; procedure codes O7020, O7061, O7062 | Any / inpatient or outpatient |
| Cardiac surgery | Procedure codes  PCI: O1640-O1649, OA640-OA642, OA647-OA649  CABG: M6551, M6552, M6561-M6564, M6567, M6571, M6572  Aortic valve procedure: O1781-O1783, O1791-O1799, M6531-M6533  Pacemaker and/or intra-cardiac defibrillation: O0203-O0210, O0211-O0214, O0219-O0222  Other cardiac surgery: O0881-O0889, O0710, O0711, O1721-O1723, O1730, O1740, O1750, O1760, O1770, O1810, O1821-O1826, O1830 | NA / inpatient or outpatient surgery |

Abbreviations: CABG, coronary artery bypass grafting; ESRD, end stage renal disease; PCI, percutaneous coronary intervention.

**Table S2. List of International Classification of Disease 10^th^ revisions (ICD-10) codes to define each outcome**

| **Outcome** | **ICD-10 code** | **Type of diagnosis (primary, secondary)/ type of hospital contact** |
| --- | --- | --- |
| Heart failure readmission | I110, I130, I132, I50x | Any / admission or emergency department visit |
| Cardiovascular death | I00.x - I77.x [excl. I46.9] | In-hospital death with a cardiovascular diagnosis |

**Table S3. List of covariates for generating propensity score**

| **Characteristics** | **Categories** |
| --- | --- |
| Age | Continuous |
| Sex | Male, Female |
| Cohort entry year | 2016, 2017, 2018, 2019 |
| **Comorbidity in past 1 year** | **ICD-10 or procedure codes** |
| Atrial fibrillation | I48x |
| Cancer (excl. non-melanoma skin cancer) | C00x-C43x, C45x-C97x |
| Cardiac surgery | Procedure codes  PCI: O1640-O1649, OA640-OA642, OA647-OA649  CABG: M6551, M6552, M6561-M6564, M6567, M6571, M6572  Aortic valve procedure: O1781-O1783, O1791-O1799, M6531-M6533  Pacemaker and/or intra-cardiac defibrillation: O0203-O0210, O0211-O0214, O0219-O0222  Other cardiac surgery: O0881-O0889, O0710, O0711, O1721-O1723, O1730, O1740, O1750, O1760, O1770, O1810, O1821-O1826, O1830 |
| Cerebrovascular disease | I60x-I69x, G45x (excl G454), G46 |
| Coronary artery disease | I20x, I21x, I22x |
| Chronic kidney disease | N18x, N19x |
| Chronic liver disease | B18x, B19x, I850, I859, I982, K70x-K77x |
| Chronic respiratory disease | J84x, J41x-J47x |
| Dyslipidemia | E78x |
| Heart failure | I50x |
| Hypertension | I10x, I11x, I12x, I13x, I15x |
| Peripheral artery disease | I70x, I73x |
| **Comedications in past 1 year** | **ATC codes** |
| Antiplatelets/anticoagulants | B01AC, B01AA, B01AE07, B01AF |
| β-blockers | C07 |
| Calcium channel blockers | C08C, C08D |
| Digoxin | C01AA05 |
| Diuretics | C03C, C03EB, C03A, C03B, C03D, C03EA |
| Nitrates | C01DA |
| RASi | C09A-C09D |
| Lipid lowering drug | C10 |
| **Medication at discharge** | **ATC codes** |
| RASi | C09A-C09D |
| β-blockers | C07 |
| Mineralocorticoid receptor antagonist | C03DA |
| ARNi | C09DX04 |
| **Antidiabetic drugs  in past 1 years** | **Categories or ATC codes** |
| Level of treatment | Level 1, only one antidiabetic drug class; level 2: ≥2 different classes of non-insulin antidiabetic drugs; level 3 ≥1 insulin either alone or in combination with other antidiabetic drugs |
| DPP4i | A10BH, A10BD07, A10BD08, A10BD09, A10BD10, A10BD11, A10BD13, A10BD18, A10BD19, A10BD21, A10BD22, |
| GLP-1RA | A10BJ |
| Insulin | Fast-acting: A10AB, A10AD  Intermediate- or long-acting: A10AC, A10AD, A10AE |
| Metformin | A10BA02, A10BD02, A10BD03, A10BD05, A10BD07, A10BD08, A10BD10, A10BD11, A10BD13, A10BD14, A10BD15, A10BD16, A10BD20 |
| SGLT2i | A10BK, A10BD15, A10BD16, A10BD19, A10BD20, A10BD21, A10BD23, A10BD24, A10BD25, |
| Sulfonylureas | A10BB, A10BD01, A10BD02, A10BD04, A10BD06 |
| Thiazolidinediones | A10BG, A10BD03, A10BD04, A10BD05, A10BD06, A10BD09, A10BD26 |
| Others (AGI, glinides) | A10BF01, A10BF02, A10BF03, A10BD14, A10BX |
| **Healthcare use in past 1 years** | **Categories** |
| Number of hospitalizations | 0, 1-2, ≥ 3 |
| Number of outpatient visits | 0-2, 3-5, ≥6 |

Abbreviations: AGI, α-glucose inhibitor; ARNi, angiotensin receptor-neprilysin inhibitor; CABG, coronary artery bypass grafting; DPP4i, dipeptidyl peptidase-4 inhibitor; GLP-1RA, glucagon-like peptide-1 receptor agonist; PCI, percutaneous coronary intervention; RASi, renin-angiotensin system inhibitors; SGLT2is, sodium-glucose co-transporter 2 inhibitor

**Table S4. SGLT2i treatment before and after weighting**

| **Treatment** | **Unweighted** | | **Weighted^†^** | |
| --- | --- | --- | --- | --- |
|  | **N** | **(%)** | **N** | **(%)** |
| SGLT2i | 818 | (100.0) | 26,029 | (100.0) |
| Dapagliflozin | 416 | (50.9) | 14,437 | (55.5) |
| Empagliflozin | 401 | (49.0) | 11,660 | (44.8) |
| Ertugliflozin | 4 | (0.5) | 158 | (0.6) |
| Ipragliflozin | 0 | (0.0) | 0 | (0.0) |

† Inverse probability of treatment weighting on the propensity score with asymmetrical trimming was used to balance comparison groups on indicators of baseline characteristics. This method produced a weighted pseudo sample of patients in the exposed and reference group with the same distribution of measured covariates. Propensity scores were reestimated within each subgroup.

**Table S5. Results of sensitivity analyses**

| **Analyses** | **Weighted HR^†^ (95% CI)** |
| --- | --- |
| Main analysis | 0.90 (0.87-0.93) |
| Alternative definition of outcome^‡^ | 0.90 (0.87-0.93) |
| Alternative definition of comparator |  |
| Second-line antidiabetics | 0.90 (0.87-0.93) |
| DPP4i | 0.73 (0.70-0.77) |
| Trimming thresholds 2.5 and 97.5% | 0.95 (0.91-0.98) |
| Trimming thresholds 5.0 and 95.0% | 0.90 (0.87-0.94) |
| Fine stratification (ATE) | 0.86 (0.68-1.09) |

† Inverse probability of treatment weighting on the propensity score with asymmetrical trimming was used to balance comparison groups on indicators of baseline characteristics. This method produced a weighted pseudo sample of patients in the exposed and reference group with the same distribution of measured covariates. Propensity scores were reestimated within each subgroup.

‡ Defined heart failure readmission using primary diagnoses only

Abbreviations: HF, heart failure; HR, hazard ratio; IPTW, inverse probability treatment weighting; PS, propensity score

**Figure S1. Propensity score distribution before and after applying inverse probability treatment weighting with asymmetrical trimming**
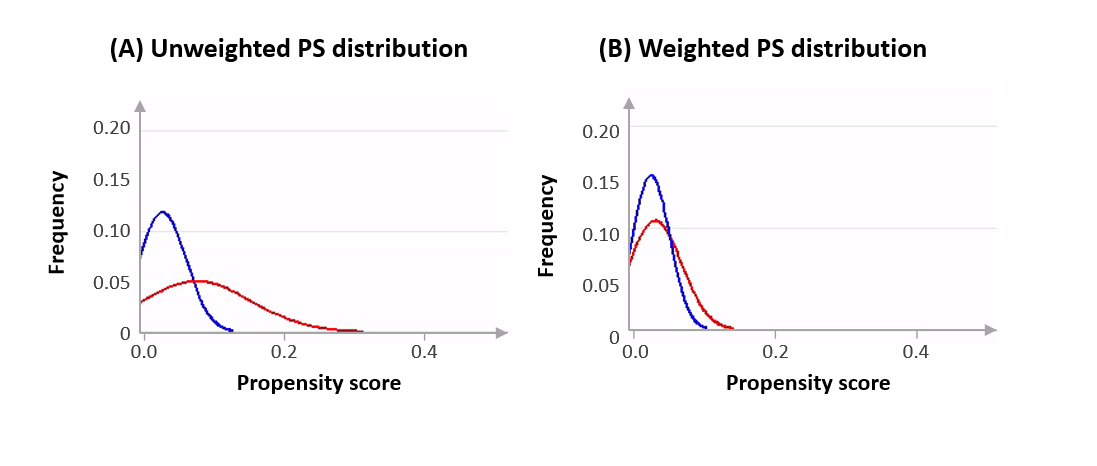


Note: Red lines indicate the distributions of estimated propensity score in exposed (sodium-glucose cotransporter 2 inhibitor) group, and blue lines indicate those of in comparator (non-use) group. Inverse probability of treatment weighting on the propensity score with asymmetrical trimming was used to balance comparison groups on indicators of baseline characteristics. We excluded patients who were treated most contrary to prediction using cutpoints corresponding to the 1th and 99th percentiles of the PS distribution in the treated and untreated patients, respectively. This method produced a weighted pseudo sample of patients in the exposed and reference group with the same distribution of measured covariates.


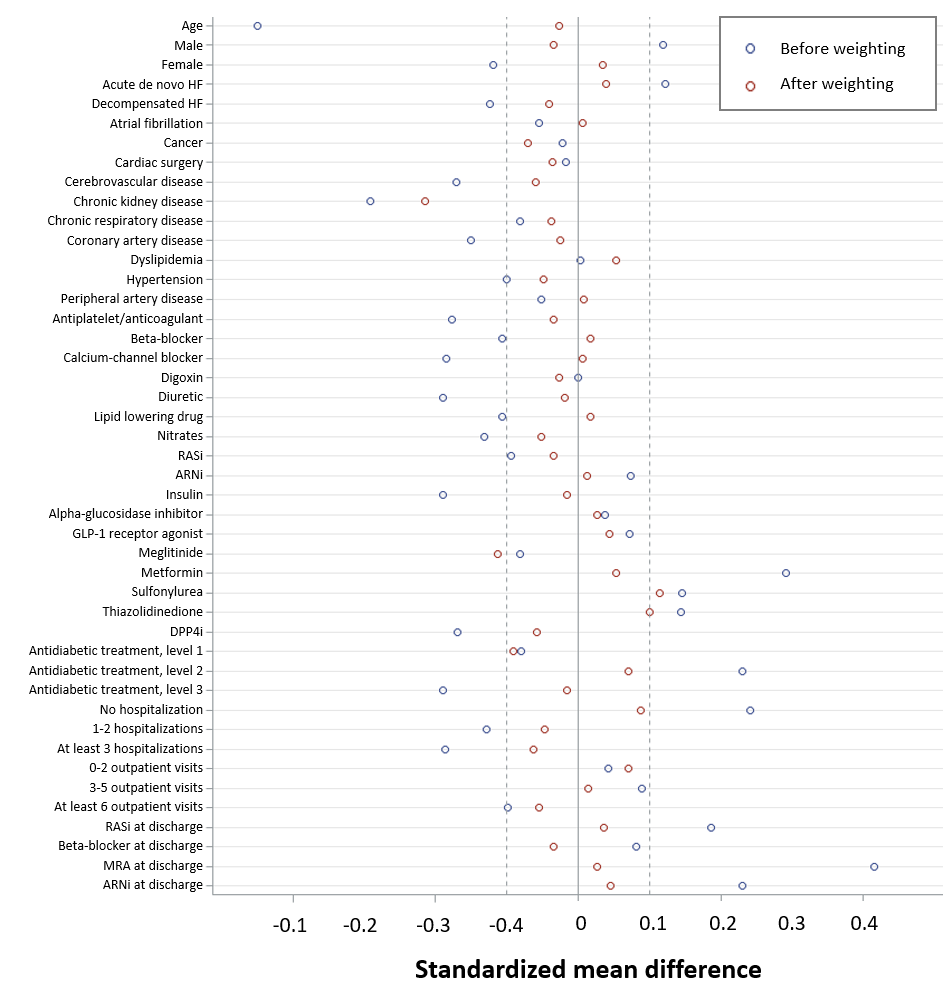


Figure S2. Plot of standardized mean differences before and after weighting

Note: Based on the propensity score, inverse probability of treatment weighting with asymmetrical trimming was used to balance exposed and comparator groups on measured covariates. This method generated a pseudo population of weighted patients in the exposed and comparator group with similar distribution of measured covariates. All covariates, except for medication at discharge, were assessed during the year before cohort entry, including the cohort entry date. Use of antidiabetic drugs past 365 days before the date of cohort entry: level 1, only one antidiabetic drug; level 2, at least two classes of noninsulin antidiabetic drugs; or level 3 at least one insulin treatment as alone or in combination with other antidiabetic drugs.

Abbreviations: ARNi, angiotensin receptor/neprilysin inhibitor; DPP4i, dipeptidyl peptidase-4 inhibitor; HF, heart failure; MRA, mineralocorticoid receptor antagonist; RASi, renin-angiotensin system inhibitor


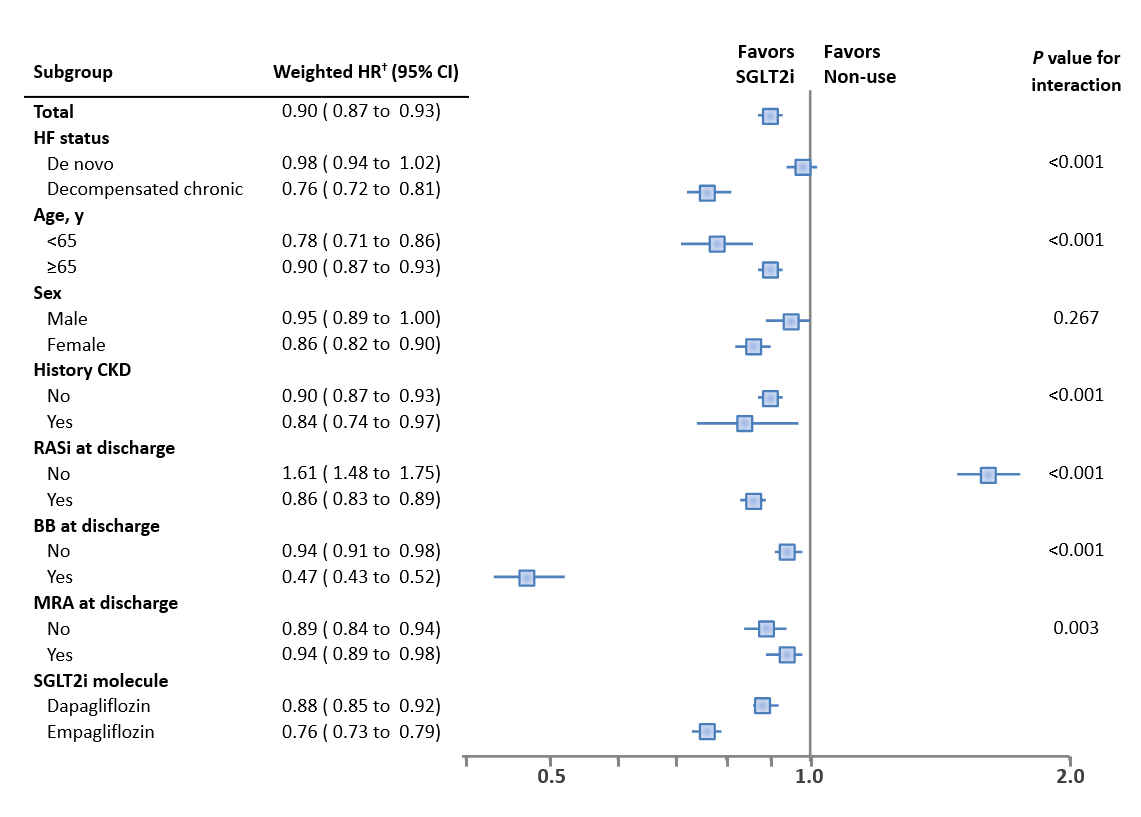


**Figure S3. Forest plot for the results of subgroup and stratified analyses on 1-year post-discharge outcome**

†Inverse probability of treatment weighting on the propensity score with asymmetrical trimming was used to balance comparison groups on indicators of baseline characteristics. This method produced a weighted pseudo sample of patients in the exposed and reference group with the same distribution of measured covariates. Propensity scores were reestimated within each subgroup.

Abbreviations: BB, β-blocker, CI, confidence interval; CKD, chronic kidney disease; CVD, cardiovascular disease; HR, hazard ratio; MRA, mineralocorticoid receptor antagonist; RASi, renin-angiotensin system inhibitor; SGLT2i, sodium-glucose co-transporter 2 inhibitor.


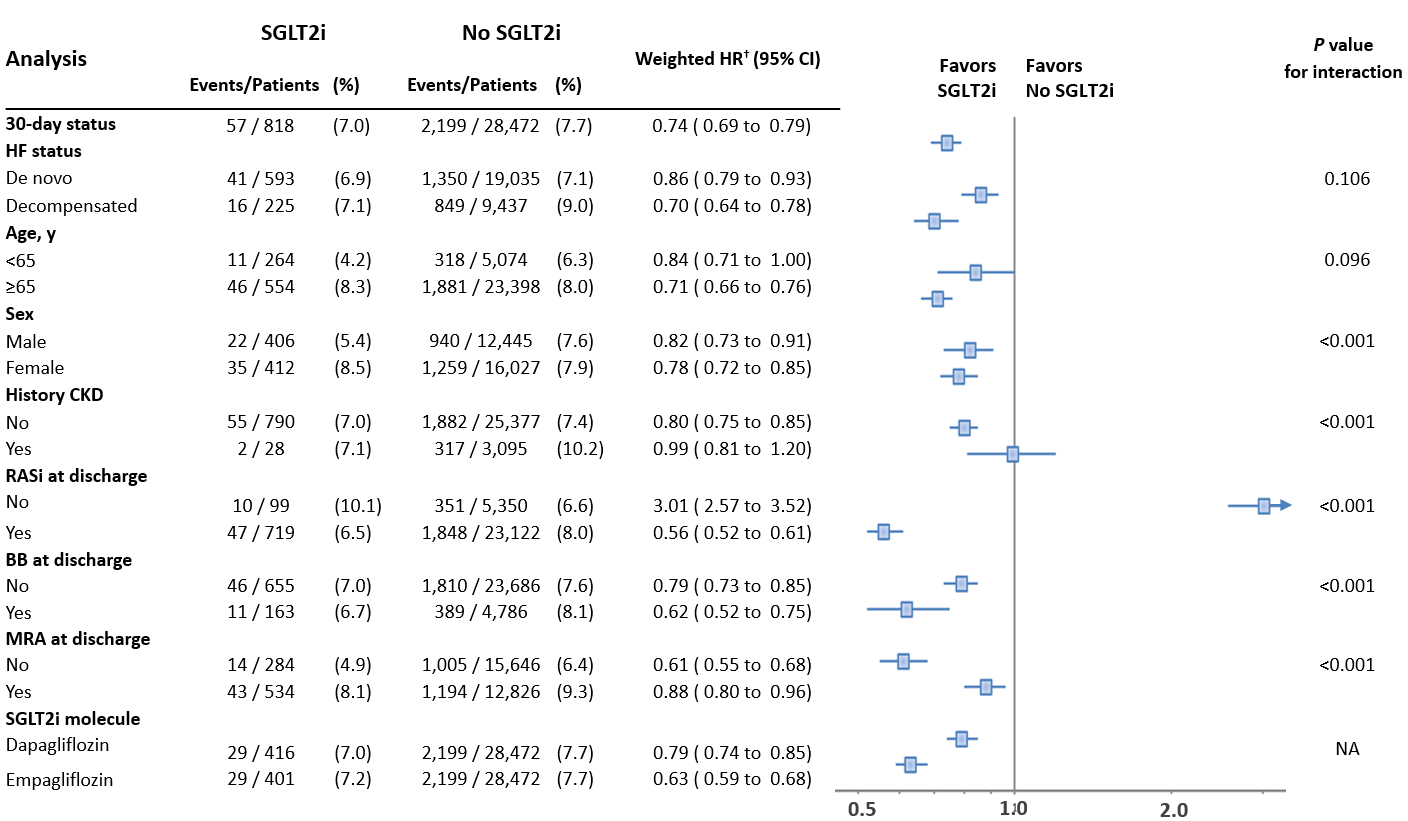


**Figure S4. Results of subgroup and stratified analyses on 30-day post-discharge outcome**

† Based on the propensity score, inverse probability of treatment weighting with asymmetrical trimming was applied to balance exposed and comparator groups conditional on measured covariates. This method generated a pseudo population of weighted patients with similar distribution of measured covariates in the exposed and comparator groups. Propensity scores were reestimated within each subgroup.

**Abbreviations:** BB, beta-blocker; CI, confidence interval; CKD, chronic kidney disease; DPP4i, dipeptidyl peptidase-4 inhibitor; HF, heart failure; MRA, mineralocorticoid receptor antagonist; RASi, renin-angiotensin system inhibitor; SGLT2i, sodium-glucose co-transporter 2 inhibitor


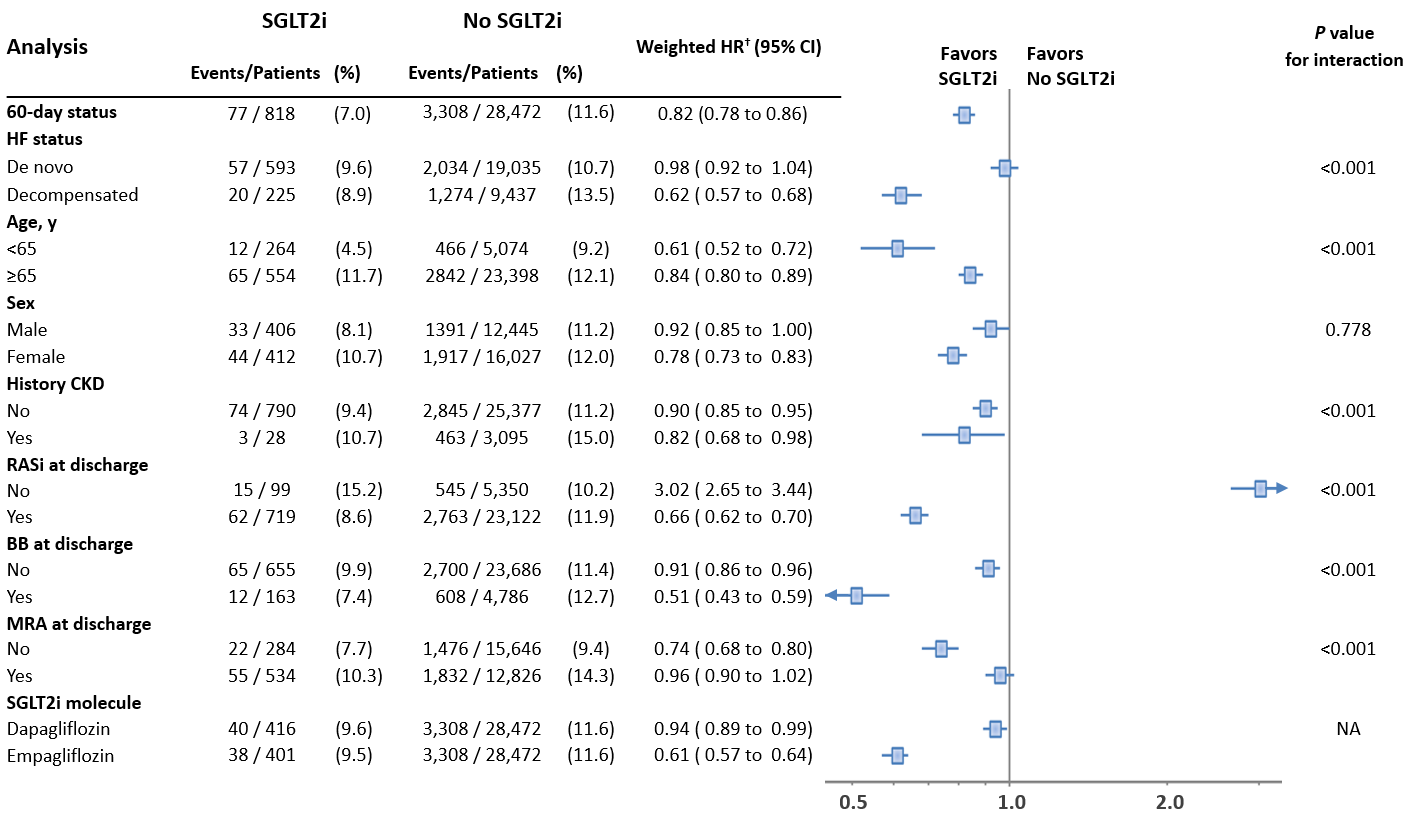


**Figure S5.** **Results of subgroup and stratified analyses on 60-day post-discharge outcome**

† Based on the propensity score, inverse probability of treatment weighting with asymmetrical trimming was applied to balance exposed and comparator groups conditional on measured covariates. This method generated a pseudo population of weighted patients with similar distribution of measured covariates in the exposed and comparator groups. Propensity scores were reestimated within each subgroup.

**Abbreviations:** BB, beta-blocker; CI, confidence interval; CKD, chronic kidney disease; DPP4i, dipeptidyl peptidase-4 inhibitor; HF, heart failure; MRA, mineralocorticoid receptor antagonist; RASi, renin-angiotensin system inhibitor; SGLT2i, sodium-glucose co-transporter 2 inhibitor


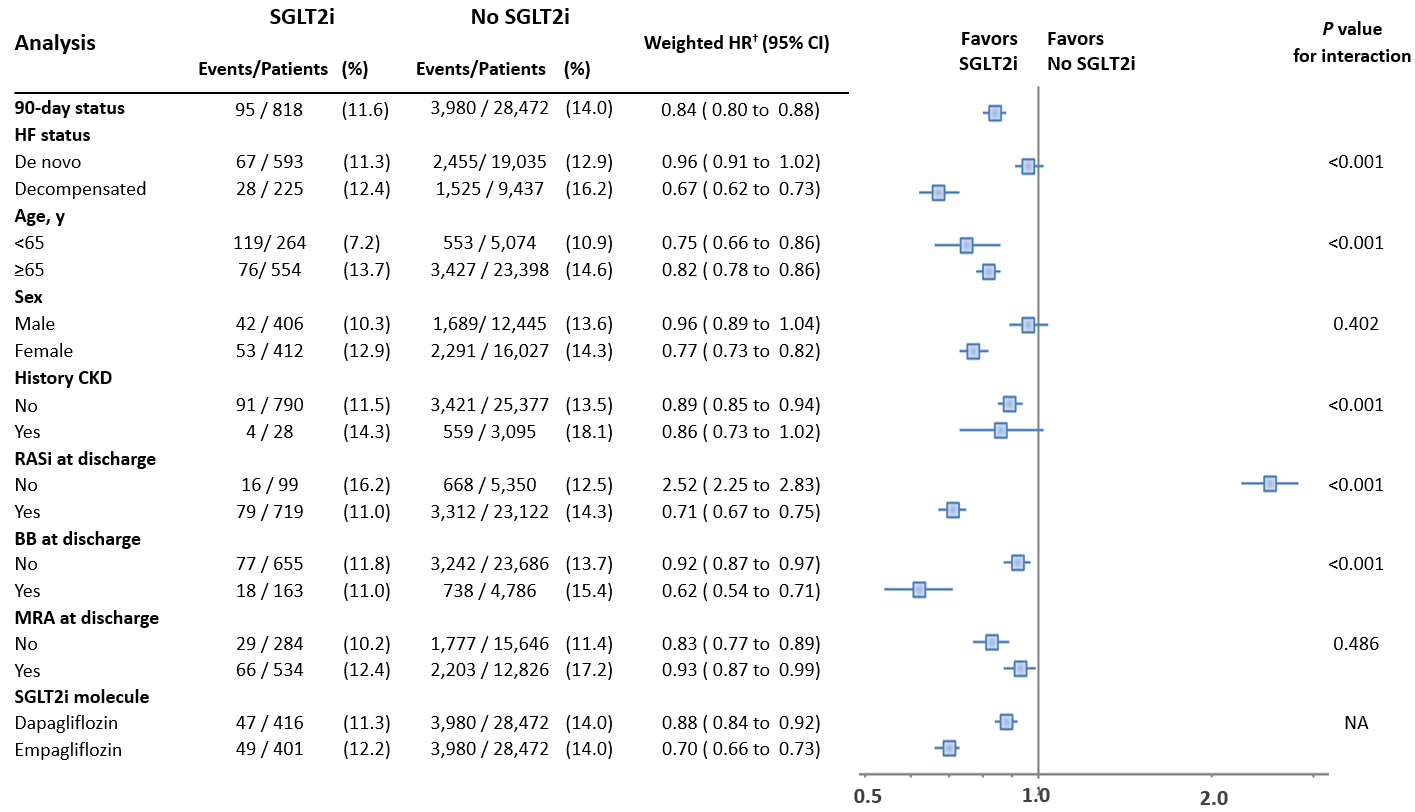


**Figure S6.** **Results of subgroup and stratified analyses on 90-day post-discharge outcome**

† Based on the propensity score, inverse probability of treatment weighting with asymmetrical trimming was applied to balance exposed and comparator groups conditional on measured covariates. This method generated a pseudo population of weighted patients with similar distribution of measured covariates in the exposed and comparator groups. Propensity scores were reestimated within each subgroup.

**Abbreviations:** BB, beta-blocker; CI, confidence interval; CKD, chronic kidney disease; DPP4i, dipeptidyl peptidase-4 inhibitor; HF, heart failure; MRA, mineralocorticoid receptor antagonist; RASi, renin-angiotensin system inhibitor; SGLT2i, sodium-glucose co-transporter 2 inhibitor
